# Backstage Heroes—Yeast in COVID-19 Research

**DOI:** 10.3390/ijms252312661

**Published:** 2024-11-25

**Authors:** Wojciech Grabiński, Andonis Karachitos, Anna Kicińska

**Affiliations:** Department of Bioenergetics, Institute of Molecular Biology and Biotechnology, Faculty of Biology, Adam Mickiewicz University, 61-614 Poznań, Poland; wojciech.grabinski@amu.edu.pl (W.G.); andonis.karachitos@amu.edu.pl (A.K.)

**Keywords:** *Saccharomyces cerevisiae*, COVID-19, SARS-CoV-2, vaccines, yeast surface display, yeast two-hybrid system

## Abstract

The extremely rapid development of understanding and technology that led to the containment of the COVID-19 pandemic resulted from collaborative efforts in the fields of *Betacoronavirus pandemicum* (SARS-CoV-2) biology, pharmacology, vaccinology, and medicine. Perhaps surprisingly, much of the research was conducted using simple and efficient yeast models. In this manuscript, we describe how yeast, eukaryotic microorganisms, have been used to research this global challenge, focusing on the therapeutic potential of the studies discussed herein. Thus, we outline the role of yeast in studying viral protein interactions with the host cell proteome, including the binding of the SARS-CoV-2 virus spike protein to the human ACE2 receptor and its modulation. The production and exploration of viral antigens in yeast systems, which led to the development of two approved COVID-19 vaccines, are also detailed. Moreover, yeast platforms facilitating the discovery and production of single-domain antibodies (nanobodies) against SARS-CoV-2 are described. Methods guiding modern and efficient drug discovery are explained at length. In particular, we focus on studies designed to search for inhibitors of the main protease (Mpro), a unique target for anti-coronaviral therapies. We highlight the adaptability of the techniques used, providing opportunities for rapid modification and implementation alongside the evolution of the SARS-CoV-2 virus. Approaches introduced in yeast systems that may have universal potential application in studies of emerging viral diseases are also described.

## 1. Introduction

Since 2019, coronavirus disease 2019 (COVID-19), an acute respiratory infectious disease caused by *Betacoronavirus pandemicum* (also known as severe acute respiratory syndrome coronavirus 2 (SARS-CoV-2)), has rapidly spread worldwide, resulting in unprecedented challenges for public health. As the infection symptoms often progress to the development of life-threatening acute respiratory distress syndrome, severe pneumonia, respiratory failure, metabolic acidosis, hypoxia, septic shock, multiple organ failure, and death, immediate and widespread preventive measures were deemed to be necessary [1].

The COVID-19 pandemic is associated with stay-at-home orders, lockdowns, and mandatory face covering. The pandemic is also remembered for the need to develop coping mechanisms, including engagement in some form of physical activity or hobbies (music, cooking, gardening, etc.). We may recall the rediscovery of baking homemade bread across the globe, which led to an immensely increased demand for the budding yeast *Saccharomyces cerevisiae*. The organisms used for millennia to make bread, beer, and wine have been indispensable in the 21st century. However, this is only a tiny part of the contribution of yeast to the battle against the coronavirus. As model organisms, yeast have proven to be instrumental for studying aspects of SARS-CoV-2 biology, rapid development of diagnostic tools, and effective vaccines and antivirals. They have even enabled the development of undergraduate course that allowed students to design and conduct CRISPR editing experiments at home during lockdown [2].

In biology research, yeast are among the most important, but often underestimated, model organisms. They have previously had a key impact on the discovery of many cellular mechanisms, such as nucleic acid metabolism, DNA repair, cell cycle regulation, gene expression, organelle biogenesis, and the response to diverse stressors [3]. Yeast are also widely used in studies of various human diseases, including mitochondrial, neurodegenerative, and metabolic disorders [4,5,6]. The advantages of using yeast in research include its low cost, simple growth conditions, rapid growth, and lack of existing ethical issues. However, yeast are also unicellular eukaryotic organisms, with relatively small genomes incorporating few gene duplications, and are easily manipulated with various molecular and genetic methods, making them suitable for the application of genome engineering techniques, including the CRISPR/Cas9 gene editing tool. The proteins expressed in yeast cells are subject to posttranslational modifications characteristic of eukaryotic cells and are suitable for flow cytometry, which allows quantitative measurements without soluble protein expression and purification [7].

Here, we present a comprehensive overview of yeast-based research that has not only advanced our understanding of coronavirus biology but also led to the development of potent vaccines and therapeutics.

## 2. Overview of the Most Common Yeast Techniques Used in COVID-19 Studies

The manufacturing of many biological drug substances and industrial enzymes, such as phytases, lipases, mannanases, and xylanases, involves recombinant protein production technology [8]. Because of their ability to secrete proteins, their heterologous protein productivity, and their economic advantages, yeast are frequently used for protein expression. *Komagataella phaffii* (*Pichia pastoris*), for example, offers high protein folding efficiency, high-cell-density fermentation, a strong and highly regulated expression system, genetic stability, robust protein secretion, and easy downstream processing [9]. The selection of constitutive and inducible promoters can be used, as can improved secretion signals [10], coexpression of chaperones [11], humanization of glycosylation [12,13], and protease-deficient strains [14].

In addition, several protein engineering tools have been developed and optimized in yeast. In the next paragraphs, we briefly describe the techniques most commonly applied in COVID-19 research.

**Figure 1:**
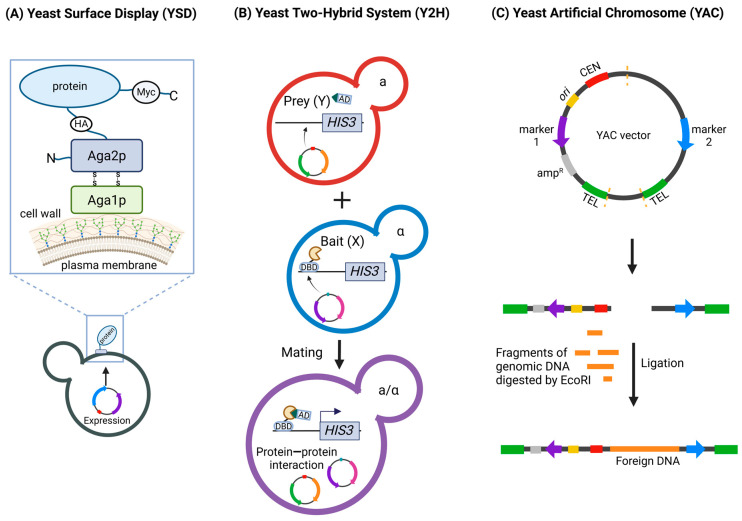
Overview of Yeast-Based Techniques Used in COVID-19 Research. (**A**) Yeast Surface Display (YSD). YSD allows the display of recombinant proteins on the surface of *Saccharomyces cerevisiae*. The figure shows the protein of interest linked to Aga2p, which is attached to the cell wall via disulfide bonds with Aga1p. (**B**) The Yeast Two-Hybrid (Y2H) system. Y2H is used to study protein—protein interactions. The figure depicts two haploid yeast cells, each expressing a different fusion protein: Bait (X) with a DNA-binding domain (DBD) and Prey (Y) with an activation domain (AD). Upon mating, the X and Y interact, bringing the DBD and AD into proximity and leading to the activation of a reporter gene (HIS3). (**C**) Yeast Artificial Chromosomes (YACs). YACs are vectors used to clone large DNA fragments. The figure shows the construction of a YAC by digesting the vector, followed by ligation with foreign DNA. MYC—a Myc-tag, HA—hemagglutinin-tag, TEL—telomere, CEN—centromere. Figure created with BioRender.com.

Yeast surface display (YSD) allows the display of recombinant proteins on the surface of *Saccharomyces cerevisiae* cells via genetic fusion to an abundant cell wall protein [15,16]. As it allows proper folding and modification of expressed heterologous eukaryotic proteins, YSD has proven to be a very valuable tool for studying protein interactions, as well as for the development of vaccines and therapeutics, including nanobodies, against SARS-CoV-2. Although yeast offers multiple options for cell surface anchor proteins, we briefly introduce an a-agglutinin system as an example (Figure 1A). A-agglutinin (mating-type-specific agglutinin) consists of a subunit encoded by AGA1 and linked through disulfide bonds to a binding subunit encoded by AGA2. The heterologous proteins are fused to the C-terminus of Aga2p [15]. The Aga2p fusion protein and Aga1p associate within the secretory pathway, are exported to the cell surface, and are covalently linked to the cell wall. The protein of interest is expressed as a fusion with two additional epitope tags: a haemagglutinin (HA) tag between Aga2p and the N-terminus of the protein of interest and a C-terminal c-myc tag. The epitope tags allow the quantification of fusion protein expression [7].

Another method, the yeast two-hybrid (Y2H) system, originally described in 1989 by Fields and Song [17], has since been successfully used to study protein—protein interactions. The architecture of many transcriptional activator proteins has been key for their discovery. These proteins have two separate domains: a transcription-activating domain (AD) and a DNA-binding domain (DBD) [18]. To function, these domains must be in close contact spatially, but they do not have to be part of the same protein. Thus, in the Y2H method, yeast are transformed with two separate plasmids containing the coding sequences of the fusion proteins DBD-X and AD-Y, where X and Y are the sequences coding for the tested proteins. If the tested proteins (X, Y) closely interact within the nucleus of yeast cells and bring BD and AD to close proximity, the activation of reporter gene transcription is observed [17] (Figure 1B). Importantly, the interaction is studied in vivo, and protein purification is not needed. The system has been modified and has since been used for numerous applications, including library screening, the study of RNA—protein interactions, and the identification of membrane-bound proteins [19,20,21]. This system has been used to map interactions between SARS-CoV-2 proteins and human host proteins. The Y2H method is also applied to test compounds that disturb known interactions. In these cases, reporter gene expression is toxic to cells. The tested compounds that interfere with protein—protein interactions support cell survival [22].

Yeast artificial chromosomes (YACs) were first constructed as linear 55 kb DNA molecules that maintain many properties of natural yeast chromosomes, e.g., mitotic stability and a low copy number [23]. They contain cloned genes, replicators, centromeres, and telomeres. Their potential as a cloning system for large DNA fragments has been recognized (Figure 1C) [24]. The method was used for cloning large viral genomes, including the SARS-CoV-2 genome, facilitating the study of viral genetics and the development of viral replicons. Recent advances in cloning methods have advanced this technology. A system based on transformation-associated recombination (TAR) in the yeast *S. cerevisiae*, which benefits from yeast homologous recombination mechanisms, allows recombination between the vector and homologous sequences in cotransformed DNA and enables the selective isolation of any genomic fragment or gene of interest up to 250 kb in size from complex genomes without the need to construct and screen genomic YAC libraries of random fragments. The TAR vector contains gene-specific targeting sequences (hooks) at both ends that enable highly efficient recombination with the gene of interest and result in the formation of a circular YAC molecule [25]. 

## 3. SARS-CoV-2 Interactions with Host Proteins

SARS-CoV-2 entry is initiated by the binding of the receptor-binding domain (RBD) of the spike (S) glycoprotein to the host cell receptor angiotensin-converting enzyme type II (ACE2). RBD−ACE2 binding is followed by the cleavage of the S-glycoprotein by host proteases, allowing membrane fusion [26]. A deep understanding of the mechanism of the virus—receptor interaction and its regulation is crucial for infectivity studies, the development of antibody-based therapies, and basic viral biology studies.

Yeast systems were key in discoveries leading to the thorough characterization of the SARS-CoV-2-ACE2 complex. As the improved YSD platform is one of the most widely used methods for directed protein evolution, it was frequently used in these studies [27]. For example, the combination of multiple rounds of random mutagenic libraries of the RBD and in vitro evolution to affinity-maturate the RBD by binding to decreasing concentrations of ACE2 led to the identification of RBD variants with high affinity binding to the receptor [28]. The study revealed that mutations present in more transmissible viruses (S477N, E484K, and N501Y) were preferentially selected via high-throughput screening. The evolved RBD mutants included the amino acid substitutions found in the RBDs of the B.1.620, B.1.1.7 (Alpha), B1.351 (Beta), and P.1 (Gamma) variants. Moreover, the selected high-affinity variant RBD-62 inhibits infection with SARS-CoV-2 in vitro [28]. YSD has also been used to construct an enzymatically inactive and soluble form of human ACE2 that can serve as a trap-binding RBD or spike protein with high affinity [29]. ACE2 was computationally redesigned to improve the affinity towards the RBD by up to 12-fold. Random mutagenesis and selection with the pulled YSD enabled further affinity maturation with an additional 14-fold increase in binding. The addition of the ACE2 collectrin domain and fusion to a human immunoglobulin crystallizable fragment (Fc) increased stability and avidity [29]. In another study, the authors employed YSD-directed evolution to identify conserved ACE2 mutations that increase spike binding across multiple species—Gln42Leu and Leu79Ile [30].

The constructed YSD-based platform was also used for the rapid functional characterization of RBD mutants. The expression of the folded proteins and their affinity for ACE2 were investigated [31]. The results revealed constrained regions on the surface of the RBD that may be desirable targets for vaccines and antibody-based therapeutics, as well as a substantial number of mutations that are well tolerated or even enhance ACE2 binding [31]. The system was subsequently applied to predict RBD mutations that disrupt binding with antibodies used to treat COVID-19 [32,33,34,35,36,37].

Several studies have focused on more general testing of the function of individual SARS-CoV-2 proteins systematically in yeast systems. The work of Klem et al. investigated the effects of selected SARS-CoV-2 proteins on yeast growth and function [38]. The viral proteins used in the study included NSP1, NSP2, NSP9, ORF3a, E, ORF7a, and N. The expression of viral proteins or viral proteins as fusion proteins with green fluorescent protein binding protein (GBP)–red fluorescent protein (RFP) induced no obvious growth defects. The intracellular localizations of the same viral proteins were determined and were mostly vacuolar, which is consistent with protein degradation of recombinant and misfolded proteins. However, ORF3a, N, and E partially colocalized with components of the ER-to-Golgi pathway and lipid droplets. The synthetic physical interaction (SPI) method was then used to examine the forced interactions of viral proteins with the yeast proteome. GBP-RFP fusion proteins were expressed in a yeast GFP library. The results revealed that the recruitment of the SARS-CoV-2 NSP1 protein to HOPS, a vesicle-docking complex, caused the perturbation of membrane trafficking in yeast [38]. This conclusion was consistent with the hijacking of the endoplasmic–reticulum–Golgi intermediate compartment trafficking pathway during the viral infection of mammalian cells [39]. ORF3a and ORF7a, which were stably overexpressed in yeast cells, localized to the vacuolar membrane and endoplasmic reticulum, respectively [40]. The overexpression of these proteins led to the accumulation of Atg8-specific autophagosomes. In addition, ORF3a overexpression altered Ca^2+^ homeostasis, possibly via ORF3a-mediated Ca^2+^ efflux from the vacuole [40].

In an interesting attempt to fully characterize SARS-CoV-2 proteins, a library of individual SARS-CoV-2 proteins as fusion proteins with red or green fluorescent proteins was constructed [41]. These constructs were subsequently expressed in different cell types, including yeast. *S. cerevisiae* has served as a model organism for investigating direct protein–membrane interactions or interactions with highly evolutionarily conserved proteins [41]. In yeast, Nsp10-GFP displayed a punctate pattern resembling endocytic patches and showed partial colocalization with the endocytic proteins Ede1 and Sac6. The Orf3a fusion protein was localized to the late endosomal compartment [41]. These results were in agreement with observations from mammalian cells. In contrast, the expressed Orf9b-GFP fusion protein in *S. cerevisiae* appeared entirely soluble and did not exhibit mitochondrial localizations, as previously suggested [42]. Thus, the mitochondrial localizations of Orf9b rely on a specific interaction between Orf9b and human TOM70 [41]. A global approach involving the overexpression of 29 viral proteins in the fission yeast *Schizosaccharomyces pombe* also revealed that many proteins (NSP1, NSP3, NSP4, NSP5, NSP6, NSP13, NSP14, NSP15, ORF3a, ORF6, ORF7a, and ORF7b) altered cellular proliferation and integrity and induced cell death [43]. Cell death is correlated with the activation of cellular oxidative stress [43]. In another comprehensive approach, Zhou and coauthors used the Y2H system collectively with tandem mass tag affinity purification, followed by mass spectrometry (TMT-AP–MS), as a tool to generate a binary and cocomplex SARS-CoV-2 human protein–protein interactome network with the identification of 739 interactions, of which 361 were newly described [44]. Among other systems, the Y2H system allowed the identification of an interaction between ORF3a and the human transcription factor ZNF579 [44].

Interestingly, one of the SARS-CoV-2 antigen assays was also developed, which uses YSD technology to screen in vitro libraries for affinity pairs against the SARS-CoV-2 nucleocapsid protein [45].

## 4. Vaccine Development

Although COVID-19 mRNA vaccines have been developed at remarkable speed because of previous studies on the development of nucleic acid vaccine technology and other coronavirus infections (SARS-CoV, MERS), studies on more established approaches have continued [46]. Whole-inactivated virus vaccines and several recombinant protein vaccine candidates have been developed [47,48]. Some of these studies used expression in fermenting yeast as a relatively simple and cost-effective antigen protein source, which has been successfully used in middle-income countries for decades [49,50].

*P. pastoris* cultures have been used to express the SARS-CoV-2 RBD219-N1C1-spike protein receptor-binding domain, which has been modified to minimize yeast-derived hyperglycosylation and optimize yield, purity, and stability [51,52,53]. The antigen formulated on Alhydrogel^®^ has been shown to induce virus-neutralizing antibodies in mice [52]. The effect was further potentiated by the use of CpG 1829 oligodeoxynucleotide, a TLR9 agonist, for both the Wuhan isolate and other SARS-CoV-2 variants of concern [54]. Antigens produced in *P. pastoris* have become components of the Corbevax and IndoVac vaccines, and they have been administered nearly 100 million times [55]. 

Yeast-expressed proteins have also been found to show cross-effective immunity against variants of concern when wild type/delta, wild type/beta, beta/omicron, or delta/omicron RBDs are used in bivalent vaccines [56,57,58]. Other studies have shown that the expression of the RBD as a fusion with the *S. cerevisiae* a-agglutinin mating protein Aga2p subunit (as described in the YSD method) results in the presentation of the antigen on the surface of genetically modified yeast cells [59]. In addition, such vaccines can be administered orally and induce humoral and mucosal responses in mice [59,60]. Yeast *S. cerevisiae* β-glucan particles with diABZI adjuvants have also been used as RBD-based vaccine delivery systems to promote strong immune responses against SARS-CoV-2 variants in mice [61]. Glucan particles serve as pathogen-associated molecular patterns that are specifically recognized by PRR dectin-1. After internalization and cross-presentation by dendric cells, glucan particles boost mature dendric cell stimulation and drive T cell clonal expansion [62]. diABZI is a stimulator of the interferon genes (STING) agonist that induces a type I interferon response and inflammatory cytokines [63].

## 5. Antibody Discovery and Testing

In addition to the rapid development of small-molecule antiviral pharmaceuticals, the pandemic has encouraged the use of antibody-based passive immunotherapy with anti-spike protein monoclonal antibodies [64]. Monoclonal antibodies produced in mammalian cells and delivered intravenously have been shown to be effective and have been approved under emergency-use authorization [65]. At the end of this chapter, we provide some examples of how studies in yeast systems have helped to improve the monoclonal antibody repertoire. However, monoclonal antibody production is extremely expensive and prone to viral baseline resistance and treatment-emergent resistance when used as a monotherapy [66]. In contrast, single-domain antibodies (sdABs), known as nanobodies, can be produced in bacteria or yeast and may enable aerosol delivery.

**Figure 2:**
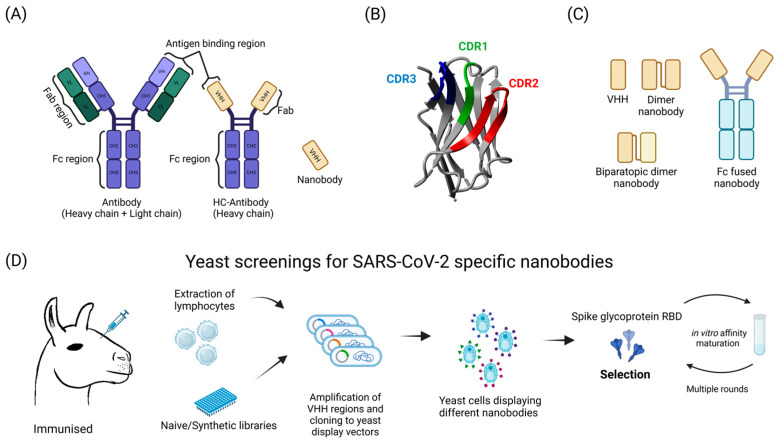
Structure and discovery of SARS-CoV-2 specific nanobodies using yeast systems. (**A**) Antibodies. A conventional antibody consists of two heavy chains and two light chains, with antigen-binding regions located on the F_ab_ (antigen-binding fragment) region. The F_c_ (crystallizable fragment) region is responsible for immune system activation. Unlike conventional antibodies, heavy-chain-only antibodies (HC-antibodies) lack light chains. Nanobodies consist only of the antigen-binding region (VHH). (**B**) Nanobody structure. The nanobody is a small, single-domain antibody. It contains three complementarity-determining regions (CDR1, CDR2, CDR3) responsible for antigen recognition and binding. The 3D structure of the nanobody was obtained from the Protein Data Bank (PDB) with the structure ID 6OBC. The visualization was created using the Yasara software, Version 20.8.15 (www.yasara.org). (**C**) Nanobody types. VHH—a single nanobody unit. Dimer nanobody—two nanobodies linked together, potentially increasing binding affinity. Biparatopic dimer nanobody—a dimer formed by two nanobodies that bind different epitopes on the same antigen. F_c_-fused nanobody—a nanobody fused to an F_c_ region, combining the small size and specificity of nanobodies with the effector functions of conventional antibodies. (**D**) Discovering SARS-CoV-2 specific nanobodies using yeast display systems. The process begins with the immunization of camelids, such as llamas, which leads to the production of heavy-chain-only antibodies (HC-antibodies). Lymphocytes are then extracted from these animals, and the VHH regions, responsible for antigen binding, are amplified and cloned into yeast display vectors, creating libraries of nanobodies. Alternatively, the synthetic or naive nanobody libraries can be used. Yeast cells are transformed to display various nanobodies on their surface. The displayed nanobodies are then screened for their ability to bind to the receptor-binding domain (RBD) of the SARS-CoV-2 spike glycoprotein. Selected nanobodies undergo in vitro affinity maturation, involving multiple rounds of selection, to enhance their binding affinity to the antigen, resulting in highly specific nanobodies against SARS-CoV-2. Figure created with BioRender.com.

Nanobodies are small (~15 kDa), water-soluble, and stable proteins that bind their targets with high specificity and affinity [67]. They are derived from heavy-chain-only antibodies (HC-Abs) that are naturally present in the Camelidae family. Nanobodies are, in fact, the isolated HC-Ab terminal variable domain VHH, which contains three complementarity-determining regions (CDR1-3) responsible for antigen recognition (Figure 2) [68]. In an attempt to further improve their capacity, dimeric, biparatopic, and Fc-fused nanobodies were used. To date, an assortment of sdAbs against SARS-CoV-2 have been developed. Most of them target the receptor binding domain (RBD) of the spike glycoprotein, and many of them have been studied in yeast systems [69]. Some examples are described below.

A platform for in vitro nanobody discovery with the aid of modified YSD in *S. cerevisiae* was developed in 2018 [70]. A synthetic nanobody library was constructed on the basis of a consensus sequence based on Ilama (*Lama glama*) genes with designed variations in the CDR loops that comprise the highly variable, antigen-binding interface of the single variable domain (VHH). The applied YSD was simplified by replacing the canonical Aga2p—Aga1p linkage to the cell wall with a single protein tether. The library enabled the expression of at least 108 unique full-length nanobodies, which are displayed on the yeast cell surface [70] (Figure 2D). The library was initially screened for binders of the SARS-CoV-2 spike ectodomain, and sdAbs were selected by further rounds of binding. This resulted in the selection of Nb6, which binds to the spike while the spike protein is in a fully inactive conformation and is incapable of binding ACE2. Affinity maturation and structure-guided design yielded a trivalent nanobody, mNb6-tri, with femtomolar affinity for spike and the picomolar neutralization of SARS-CoV-2 infection. mNb6-tri is stable and enables aerosolization, lyophilization, and heat treatment [71]. The modified system enabled simple directed evolution for generating nanobodies with high affinities and neutralizing activities against SARS-CoV-2 in *S. cerevisiae* [72]. The nanobody-presenting yeast were initially sorted against soluble biotinylated SARS-CoV-2 RBDs, followed by rounds of FACS RBD-Fc sorting. The leading clones were then affinity matured using error-prone PCR at a low mutational frequency. Mature nanobodies potently neutralize both SARS-CoV-2 pseudovirus and live virus and possess drug-like biophysical properties [72]. The same synthetic nanobody YSD library was used to test the ability of the newly developed autonomous hypermutation yeast surface display (AHEAD) system to generate anti-RBD nanobodies. The system combines an orthogonal error-prone DNA polymerase that replicates a specific cytosolic plasmid (p1) with yeast surface display (YSD) to achieve rapid antibody evolution through the simple cultivation and sorting of yeast cells [73]. This study resulted in the rapid generation of potent antibodies [73]. Monomeric biparatopic antibodies were also found to be potent viral neutralizers and had an enhanced ability to prevent viral escape [74] (Figure 2).

In another study, using yeast only as a straight-forward and rapid system to assist the nanobody discovery process, a combination of structure-guided molecular modeling with YSD prototyping in *P. pastoris* resulted in an affinity-enhanced VHH-human immunoglobulin G1 Fc fusion molecule with neutralizing activity [75]. YSD was also used to expand the nanobody repertoire in immunized libraries by immunizing the llamas, creating immune libraries, displaying nanobodies on yeast cell surfaces, and finally selecting new nanobodies with high affinities and neutralization activities against different SARS-CoV-2 variants [76].

In 2018, a modification of YSD that allowed the cloning of natively paired heavy- and light-chain antibody-variable regions into yeast display for functional screening was described [77]. This enabled research on human antibody variable regions in yeast systems. In one of these studies, donor peripheral blood mononuclear cells were used to investigate the involvement of paired heavy- and light-chain signatures in potent SARS-CoV-2 neutralization [78]. Later, screening the donor natively paired VH:VL at low pH in a yeast system was shown to be beneficial and led to the identification of more potent neutralizing antibodies [79]. The combination of natively paired antibody yeast display screening and coronavirus spike antigen probe panels also enabled following the responses of convalescent COVID-19 patients against continually evolving SARS-CoV-2 variants and other broadly recognized betacoronaviruses [80].

Additionally, a naive human single-chain variable fragment (scFv) antibody library incorporating a selection of antibody scaffolds (VH, VL, JL, and JH) was successfully used to generate a surface-display library in *S. cerevisiae* [81]. Later, after rounds of in vitro maturation, this library led to the discovery of three neutralizing antibodies targeting the SARS-CoV-2 RBD [81].

In a slightly different approach, YSD with a library of RBD mutants was used in studies aiming to characterize the memory B-cell repertoire of COVID-19 convalescent donors by analyzing their RBD- and non-RBD-neutralizing antibodies. The authors defined an interesting panel of potent RBD and N-terminal domain antibodies [82].

Finally, in addition to possible therapeutic and research applications, antibody discovery is a key methodology for diagnostic purposes. Among others, an adaptable serological test based on YSD, which characterizes immune profiles against SARS-CoV-2 variants of concern, has also been developed [83].

## 6. Mpro Activity Analysis

The main protease, also known as 3C-like protease (Mpro, 3CL-pro, NSP5), has drawn attention as one of the most compelling SARS-CoV-2 proteins. It is a homodimeric cysteine protease with specificity towards the peptide bonds between glutamine and serine, alanine, or glycine [84,85,86]. This key enzyme, which is involved in the virus replication cycle, cleaves pp1a and pp1ab polyproteins, which are translated from viral RNA at 11 sites, to release functional viral proteins. Importantly, Mpro itself is also released by autocatalytic cleavage between the NSP4 and NSP5 of pp1a and pp1ab [87]. Mpro is one of the most promising anti-SARS-CoV-2 drug targets, as it is essential for virus propagation, and there are no human proteases with analogous characteristics. Previous studies on other viruses, such as HIV and HCV, have suggested that viral proteases are relatively conserved [88]. Additionally, high similarity between the major proteases of different coronaviruses was observed. Thus, these inhibitors are likely broad-spectrum medicines against coronavirus infections [89,90].

Several studies have led to the identification of Mpro inhibitors, the determination of mutation effects on enzyme function, and the elucidation of the interaction of Mpro with the host cells (for a recent review, see [91,92]). A number of these studies involved the use of yeast systems and are described below.

**Figure 3:**
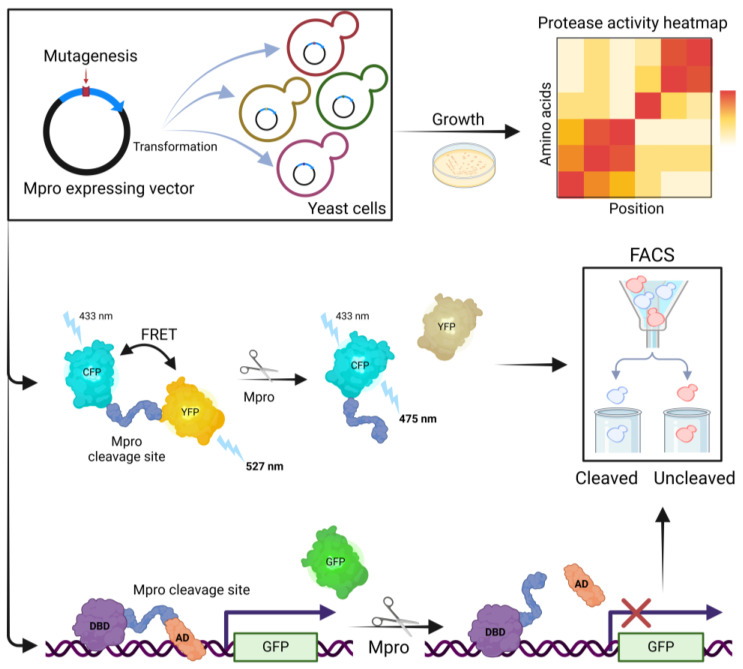
Yeast-based assays for SARS-CoV-2 main protease (Mpro) activity and mutagenesis studies. An Mpro-expressing vector undergoes mutagenesis to introduce various mutations in the protein. The mutated vectors are then transformed into yeast cells, resulting in yeast strains with different Mpro variants. Yeast cells expressing different Mpro variants are grown on selective media (**top right**). The growth rates are used to generate a heatmap, indicating protease activity across different amino acid positions and mutations. The FRET assay (**middle**) is used to measure Mpro activity. Mpro cleaves a substrate linking cyan fluorescent protein (CFP) and yellow fluorescent protein (YFP). The cleavage results in a loss of energy transfer from CFP to YFP, leading to a change in fluorescence emission from 527 nm (YFP) to 475 nm (CFP). The cleaved and uncleaved populations can be sorted using fluorescence-activated cell sorting (FACS). In cleavage assays using GFP (**bottom**), Mpro cleaves within a fusion protein that links a DNA-binding domain (DBD) and an activation domain (AD) of a transcription factor activating the green fluorescent protein (GFP) expression. Upon cleavage by Mpro, GFP expression is lost, indicating Mpro activity. Figure created with BioRender.com.

Deep mutational scanning (DMS) was used to comprehensively describe how mutations resulting in single amino acid changes affect the protease [93,94,95,96]. These studies allowed the identification of regions crucial for protein function, as well as more variable regions that are poor drug targets. The authors from Daniel Bolon’s group published a series of papers applying three different complementary yeast systems to examine Mpro activity (Figure 3) [93,94,95]. The first of these uses fluorescence resonance energy transfer (FRET). Mpro variant activity is observed in *S. cerevisiae* by measuring the decrease in fluorescence resulting from the cleavage of a YFP-CFP FRET pair linked by a sequence that natively separates NSP4 and NSP5. The second system is designed to monitor protease activity by observing GFP fluorescence. The active enzyme inactivates the transcription factor regulating GFP expression. Finally, the third system is based on Mpro toxicity in yeast cells, which is associated with limited yeast growth due to the cleavage of yeast proteins [95]. The results of all three screens were correlated and revealed 24 positions with low mutational tolerance. These included residues contacting the substrate at the dimer interface and at the interface of Domain II and Domain III, and residues being a part of an allosteric communication network between the active site and the dimerization interface [95]. The impact of possible new variants of Mpro on resistance to clinically approved nirmatrelvir and ensitrevir was also examined with this system. The identification of 99 mutations that cause resistance to both drugs could help to monitor drug resistance in circulating viral populations [94]. Further analysis of hyperactive mutations (increasing Mpro activity) revealed that these mutations are likely to contribute to the natural evolution of drug resistance in Mpro [93].

A DMS-based approach in a yeast system was also presented by Iketani et al. to systematically profile the activity of all possible single Mpro mutants [96]. In this system, the determination of Mpro activity was based on the toxicity of the active enzyme to yeast cells. Similar to previously described studies, these studies revealed nonmutable amino acid residues and other protein regions that are malleable [96].

In another notable approach, a high-copy-number plasmid-based yeast system was constructed to determine the toxicity of Mpro and other NSPs and structural SARS-CoV-2 proteins. The expression of M, E, N, NSP7, NSP8, or NSP12 resulted in no visible phenotype, whereas growth was significantly impaired in the presence of Mpro, spike glycoprotein, and the helicase [97]. The growth of Mpro-expressing yeast was restored by nirmatrelvir. Mpro variants with a mutated E166 residue were also studied. Nirmatrelvir resistance was confirmed for E166R, and reduced Mpro activity was observed for E166N in a yeast system [97].

Yeast systems have also been developed to enable drug development, with high-throughput screenings searching for Mpro inhibitors in vivo [98,99].

**Figure 4:**
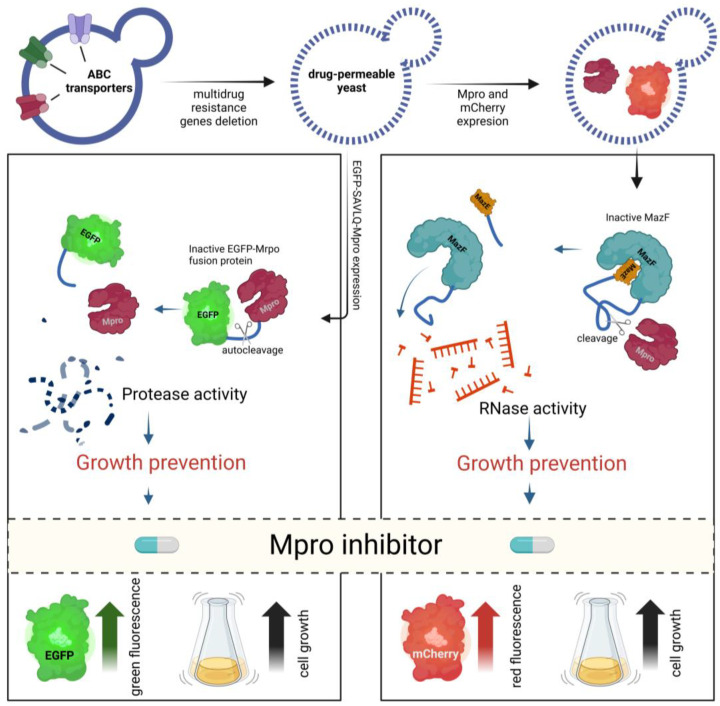
Yeast-based systems for screening SARS-CoV-2 main protease (Mpro) inhibitors. Yeast strains are genetically modified by the deletion of multidrug resistance genes, creating drug-permeable yeast suitable for high-throughput screening. Yeast cells express a fusion protein consisting of enhanced green fluorescent protein (EGFP) and Mpro, linked by a specific cleavage site (SAVLQ). Active Mpro cleaves the fusion protein, separating EGFP from Mpro, which prevents the growth of yeast cells. In the presence of an effective Mpro inhibitor, cleavage does not occur, resulting in green fluorescence and restored yeast cell growth, indicating the inhibition of Mpro activity (**left panel**). Yeast cells express the bacterial toxin MazF linked to the Mpro cleavage site and tagged with mCherry, a red fluorescent protein. Active Mpro cleaves the fusion protein, releasing MazF, which is an RNase that degrades RNA, leading to yeast growth inhibition. When an Mpro inhibitor is present, cleavage does not occur, preventing MazF release and allowing yeast cell growth. The yeast cells exhibit red fluorescence if the inhibitor is effective, signifying the prevention of RNase-induced cell death (**right panel**). Figure created with BioRender.com.

We proposed a unique system in which the Mpro-encoding gene was inserted directly into the *S. cerevisiae* genome, into chromosome 2, under the GAL1 inducible promoter, allowing controlled galactose-induced expression [99]. Additionally, the autocatalytic properties of Mpro are vital to system function. Mpro is translated as a fusion protein with EGFP linked with SAVLQ, the short sequence recognized by Mpro. After translation, Mpro cleaves the linker sequence, leading to free EGFP and Mpro proteins (Figure 4). Notably, this results in Mpro with a native N-terminal sequence without the addition of methionine, which is often used at the beginning of the Mpro protein in other expression systems. The EGFP-Mpro fusion protein shows no toxicity to yeast cells, as demonstrated with the noncleavable mutant fusion protein EGFP-D4K-Mpro [99]. Cleavage of EGFP-SAVLQ-Mpro results in the inhibition of yeast cell growth. In addition, the fluorescence level of EGFP can be monitored simultaneously. When active Mpro is present in cells, the fluorescence levels are reduced. This system enables the rapid and cost-effective screening of Mpro inhibitors. In the presence of the inhibitor, fluorescence levels increase, and yeast cell growth is unaffected. Remarkably, screening of the FDA-approved drug library led to the first identification of meisoindigo as a potent Mpro inhibitor [99]. These results were recently confirmed by Gao et al. [100].

A study by Alalam et al. used a toxin—antitoxin system derived from the E. coli protein MazF, a single-strand endoribonuclease, and MazE (a protein that binds and inhibits MazF) (Figure 4). In this system, a fusion protein consisting of MazE and MazF—MaZEF—with an Mpro-recognized linker sequence is expressed in *S. cerevisiae* [98]. The fusion protein is nontoxic. Active Mpro releases MazF molecules, which are toxic to cells, resulting in impaired growth [98]. As yeast cells are additionally tagged with the fluorescent protein mCherry, growth can be monitored via fluorescence measurements for large-scale screens [98]. Interestingly, in the system described, Mpro shows a significantly reduced toxicity to yeast cells, in contrast to previously described results [95,96,97,99].

## 7. Synthetic Recombinant Viruses and Viral Replicons

In vivo models of viral pathogenesis, as well as research into diagnostic tools, antivirals, and vaccine development, benefit immensely from reverse genetic approaches. However, the cloning of large viral genomes in bacteria is difficult, and other platforms need to be developed. Thus, with the aid of TAR cloning, yeast-based platforms have been established to obtain synthetic recombinant SARS-CoV-2 viruses or viral replicons [101,102,103]. In these studies, the viral genome is generally divided into overlapping fragments, and yeast cells are transformed with TAR vectors containing these fragments. The fragments subsequently assemble in the yeast cell to form a YAC.

The first paper by Thi Nhu Thao describes a yeast-based, fully functional synthetic genomics platform for the genetic reconstruction of various RNA viruses, including members of the Coronaviridae, Flaviviridae, and Pneumoviridae [101]. The SARS-CoV-2 genome was cloned in 12 fragments and reassembled into YAC. The isolation of the YAC’s DNA, in vitro transcription, and electroporation of BHK-21 cells with the obtained RNA enabled infectious virus isolation [101].

TAR cloning was also used to obtain viral replicons, thus avoiding the need for experiments at biosafety level 3 facilities [102]. Viral replicons are noninfectious self-amplifying nucleic acids, containing all the elements required to synthesize viral RNA but lacking structural proteins and thus not able to produce infectious viral particles [102]. The design SARS-CoV-2 replicon included the CMV promoter, the replicase gene ORF1ab, and sequences of untranslated regions (UTRs). As the viral NSP1 protein inhibits host gene expression, the replicons with NSP1 deletion were obtained [104]. In addition, a gene encoding a fusion protein consisting of turboGFP and blasticidin deaminase (GFP-BlaR) replaced the S glycoprotein coding gene [102]. 293 T cells transfected with replicon-coding plasmids showed turboGFP and viral protein expression 36 h post-transfection. Thus, the replicon could replicate and be transcribed in transfected cells. These SARS-CoV-2-GFP replicons were sensitive to previously described coronavirus replication inhibitors E64-D and remdesivir [102]. Remdesivir inhibited turboGFP expression in SARS-CoV-2-GFP replicon-transfected 293 T cells and exhibited an obvious dose dependence [102]. Similar replicons were generated by Ricardo-Lax and coauthors [103], including SARS-CoV-2 replicons where the S glycoprotein coding gene was replaced with neomycin resistance (NeoR) and a reporter gene (nuclear-localized monomeric NeonGreen or secreted Gaussia luciferase) [103]. Replicon-driven luciferase expression in transfected cells was sensitive to remdesivir, with median inhibitory concentration values similar to those previously reported for the live virus. The sensitivity to masitinib (a proposed 3C-like protease inhibitor) was also similar to values shown for infectious SARS-CoV-2 [103]. Moreover, this study indicated that replicons can be used to monitor the effects of mutations within the viral genome. The variant with a mutation impairing NSP1 gene function remained sensitive to remdesivir but was less toxic to yeast cells. Furthermore, NSP1 impairment led to hypersensitivity to interferon-α and interferon-β compared with WT replicons [103]. In addition, the replicon delivery method involves single-cycle virions that can infect cells in a spike-dependent manner but do not produce infectious progeny capable of further spread. For host cells that do not express sufficient levels of ACE2 or TMPRSS2, an alternative delivery of replicons with the vesicular stomatitis virus (VSV) glycoprotein has been developed [103].

## 8. Conclusions and Perspectives

Yeast, particularly *Saccharomyces cerevisiae* and *Pichia pastoris*, have played a major role in COVID-19 research, contributing to the understanding of virus biology, the development of diagnostics, and the production of vaccines and antiviral therapies. Yeast surface display (YSD) and yeast two-hybrid (Y2H) systems have been key to protein interaction research and inhibitor screening, most notably in the study of the interaction of the SARS-CoV-2 spike protein with the human ACE2 receptor. The development of COVID-19 vaccines has accelerated with the production of recombinant proteins such as the RBD of the spike protein in yeast systems, resulting in vaccines such as Corbevax and IndoVac. In addition, yeast have facilitated the discovery of single-domain antibodies (nanobodies) against SARS-CoV-2, offering cost-effective therapeutic potential. Yeast-based platforms have also contributed to the development of serological assays to characterize immune responses to SARS-CoV-2, which are critical for understanding virus spread and vaccine efficacy.

These organisms have also been instrumental in drug discovery, particularly with regard to screening for inhibitors of the main protease Mpro, a key enzyme in the viral replication cycle. Yeast-based systems have been developed to monitor Mpro activity and identify potential inhibitors. These approaches have led to the selection of promising Mpro inhibitors, which are now being further investigated for their potential in the treatment of COVID-19.

Despite their significant contributions, yeast face certain limitations in all aspects of COVID-19 research. The effectiveness of the expression of complex or large SARS-CoV-2 proteins in yeast could be compromised by incorrect protein folding or their toxicity to yeast cells. The functionality and immunogenicity of the viral proteins produced in yeast are also restricted by the lack of human-like post-translational modifications, such as glycosylation. Furthermore, the complexity of the human cellular environment is not fully reflected in yeast, potentially leading to the differences in the efficacy of the inhibitors identified using yeast-based platforms. Current efforts to fully humanize genetic modules in yeast should allow us to resolve at least some of these issues [105].

In the future, insights gained from yeast-based SARS-CoV-2 research open several avenues for prospective exploration and application. Improved vaccine platforms are likely to further benefit from the optimization of yeast expression systems, leading to more efficient and scalable production of vaccine antigens. Furthermore, the successful use of nanobodies against COVID-19 suggests their potential against other viral diseases.

Finally, the economic advantages of yeast systems, including low-cost production and scalability, make them suitable for large-scale biotechnological applications. These advantages are particularly relevant for developing countries, where cost-effective solutions are essential for public health care.

In summary, yeast have emerged as backstage heroes in the global response to COVID-19. The continued development and application of yeast-based technologies holds great promise for future advances in medical and biotechnology research. The lessons learned from this pandemic will undoubtedly inform and enhance our preparedness for future infectious disease outbreaks.
